# Metabolomics Reveals Strain-Specific Cyanopeptide Profiles and Their Production Dynamics in *Microcystis aeruginosa* and *M. flos-aquae*

**DOI:** 10.3390/toxins15040254

**Published:** 2023-03-31

**Authors:** Kimberlynn McDonald, Natasha DesRochers, Justin B. Renaud, Mark W. Sumarah, David R. McMullin

**Affiliations:** 1 Department of Chemistry, Carleton University, Ottawa, ON K1S 5B6, Canada; 2 London Research and Development Center, Agriculture and Agri-Food Canada, London, ON N5V 4T3, Canada

**Keywords:** cyanobacteria, cyanopeptides, metabolomics, GNPS molecular networking

## Abstract

Cyanobacterial blooms that release biologically active metabolites into the environment are increasing in frequency as a result of the degradation of freshwater ecosystems globally. The microcystins are one group of cyanopeptides that are extensively studied and included in water quality risk management frameworks. Common bloom-forming cyanobacteria produce incredibly diverse mixtures of other cyanopeptides; however, data on the abundance, distribution, and biological activities of non-microcystin cyanopeptides are limited. We used non-targeted LC-MS/MS metabolomics to study the cyanopeptide profiles of five *Microcystis* strains: four *M. aeruginosa* and one *M. flos-aquae*. Multivariate analysis and GNPS molecular networking demonstrated that each *Microcystis* strain produced a unique mixture of cyanopeptides. In total, 82 cyanopeptides from the cyanopeptolin (n = 23), microviridin (n = 18), microginin (n = 12), cyanobactin (n = 14), anabaenopeptin (n = 6), aeruginosin (n = 5), and microcystin (n = 4) classes were detected. Microcystin diversity was low compared with the other detected cyanopeptide classes. Based on surveys of the literature and spectral databases, most cyanopeptides represented new structures. To identify growth conditions yielding high amounts of multiple cyanopeptide groups, we next examined strain-specific cyanopeptide co-production dynamics for four of the studied *Microcystis* strains. When strains were cultivated in two common *Microcystis* growth media (BG-11 and MA), the qualitative cyanopeptides profiles remained unchanged throughout the growth cycle. For each of the cyanopeptide groups considered, the highest relative cyanopeptide amounts were observed in the mid-exponential growth phase. The outcomes of this study will guide the cultivation of strains producing common and abundant cyanopeptides contaminating freshwater ecosystems. The synchronous production of each cyanopeptide group by *Microcystis* highlights the need to make more cyanopeptide reference materials available to investigate their distributions and biological functions.

## 1. Introduction

In spite of progress to better control eutrophication and cyanobacterial blooms, in many regions their frequency and magnitude are increasing [1,2,3]. Cyanobacterial blooms are primarily driven by anthropogenic nutrient inputs and a changing climate [4]. Bloom events disrupt aquatic ecosystems, diminish local economies, and release phycotoxins into waterways upon senescence [5]. Due to bloom pervasiveness, cyanobacteria and their metabolites are a concern for public health and ecosystems [2,5]. Although the conditions that promote bloom formation are broadly understood, the risks and biological functions for the majority of metabolites produced by blooms are not [6,7,8,9].

**Figure 1:**
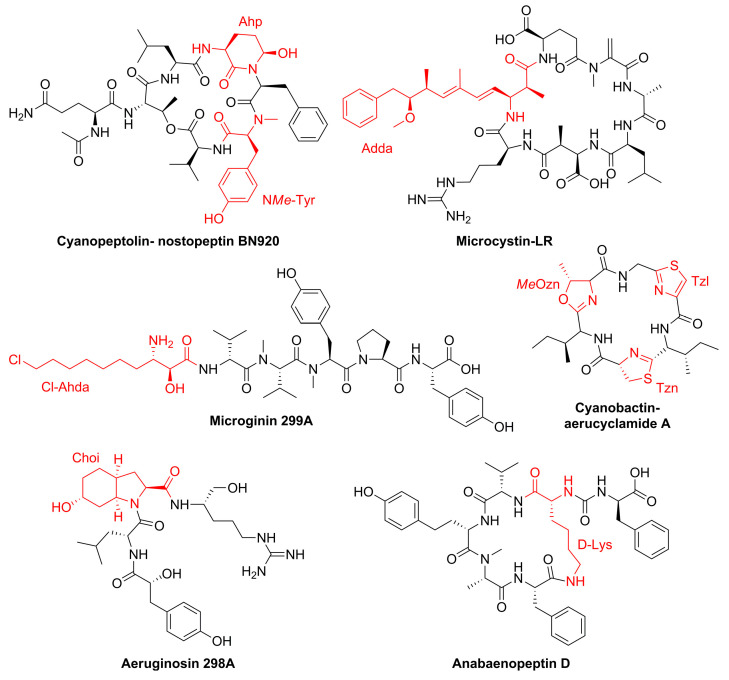
Representative cyanopeptide structures where characteristic structural features for each group appear in red.

More than 2000 cyanobacterial natural products have been described to date [10]. Cyanopeptides from non-ribosomal peptide synthetase (NRPS), hybrid NRPS–polyketide, and ribosomally synthesized and post-translationally modified peptide (RiPP) pathways account for most of this reported chemical diversity [10,11,12]. Despite this immense structural diversity, most cyanopeptide studies have focused on microcystins [13]. In addition to microcystins, common bloom-forming genera (*Microcystis, Planktothrix, Dolichospermum*, *Nodularia*) have been shown to produce additional cyanopeptide classes that have received minimal research attention, these include cyanopeptolins, aeruginosins, cyanobactins, microginins, anabaenopeptins, and microviridins [6] (Figure 1). Mining cyanobacteria genomes in public databases demonstrated that the biosynthetic gene clusters for these cyanopeptide groups, and several uncharacterized clusters, are distributed among common bloom-forming genera [14,15]. Genomic analysis of cyanopeptide biosynthetic clusters revealed that recombination events, variable NRPS adenylation domains, and flexible tailoring enzymes drastically increase the potential chemical diversity arising from these pathways [16,17]. Further, phylogenetics suggests some biosynthetic pathways may have evolved independently within each genus [15]. This indicates that our current notion of cyanobacterial natural product diversity is likely grossly underestimated.

Advancements in liquid chromatography–tandem mass spectrometry (LC-MS/MS) platforms and open-source bioinformatic tools provide opportunities to better describe microbial natural product diversity [18,19,20,21]. From cyanobacteria cultures and bloom events, metabolomic analysis has demonstrated variable co-production of multiple cyanopeptide groups and distinct profiles, even within the same species [22,23,24,25]. Microbial natural products, including cyanopeptides, are typically biosynthesized as mixtures of structurally related compounds [26]. The tandem mass spectrometry (MS/MS) spectra of chemicals are highly dependent on their molecular structure, meaning groups of structurally related compounds often generate common product ions [27,28]. Post-acquisition data processing approaches using MS/MS datasets acquired with modern high-resolution instruments, such as quadrupole time-of-flight and Orbitrap mass analyzers, can exploit these features of the natural products [28,29]. The global natural products social (GNPS) molecular networking platform is one of the most commonly used open-source bioinformatic tools for analyzing mass spectrometry datasets. Molecular networking is a non-targeted approach that compares analyte MS/MS spectra and forms clusters of putative structurally similar compounds based on shared product ions [18]. Although metabolomic tools aid natural product discovery and dereplication efforts, they do not provide unambiguous chemical characterizations nor pure compounds for further study.

In this study, we used post-acquisition non-targeted metabolomic approaches to elucidate the cyanopeptide diversity from five *Microcystis* strains: four *M. aeruginosa* and one *M. flos-aquae*. Factor analysis—principal component analysis (PCA) and factor loadings—was used on a LC-MS/MS dataset to identify cyanopeptide features that statistically contribute to metabolome variation between *Microcystis* strains. The GNPS molecular networking algorithm was subsequently used to mine MS/MS spectra to highlight cyanopeptide diversity from non-microcystin cyanopeptide groups. This non-targeted metabolomics study was performed to prioritize the cultivation of cyanobacteria strains producing target cyanopeptides for future isolation and structural characterization. The generation of analytical standards for the scientific community will help address the paucity of knowledge on the potential toxicity, biological activity, environmental concentrations, and distribution of non-microcystin cyanopeptide groups produced by bloom-forming cyanobacteria.

## 2. Results and Discussion

### 2.1. Factor Analysis of LC-HRMS Data to Reveal Cyanopeptide Variation

**Figure 2:**
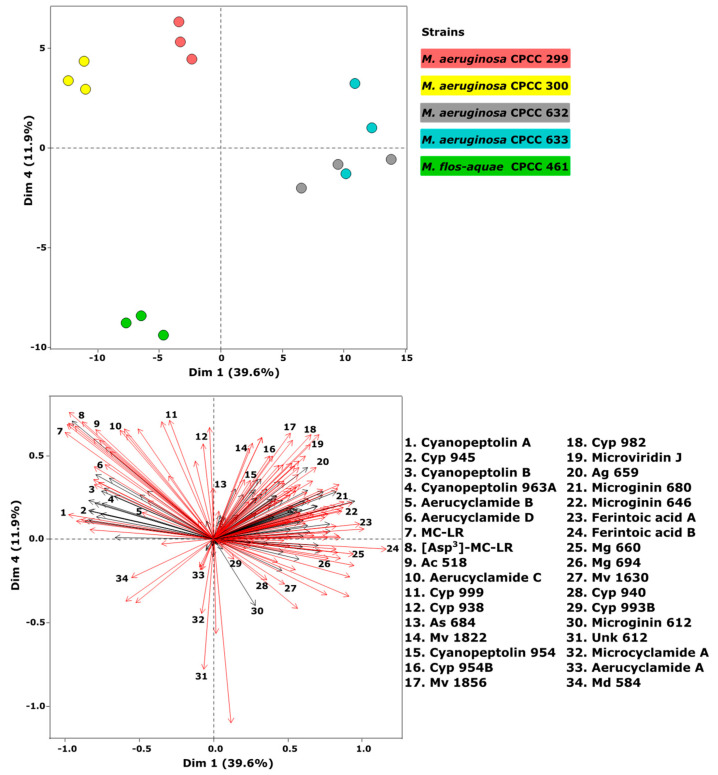
PCA (**top**) of metabolites produced by triplicate cultures of *M. aeruginosa* CPCC 299, 300, 632, and 633 and *M. flos-aquae* CPCC 461 grown in BG-11 medium. The loading plot (**bottom**) contains all factor loadings considered. A total of 262 variables are considered in the PCA and visualized in the loading plot. Red arrows indicate statistical significance in contribution to the individual strains in the PCA (*p* < 0.05 Kruskal–Wallis test with Benjamini-Hochberg correction).

**Figure 3:**
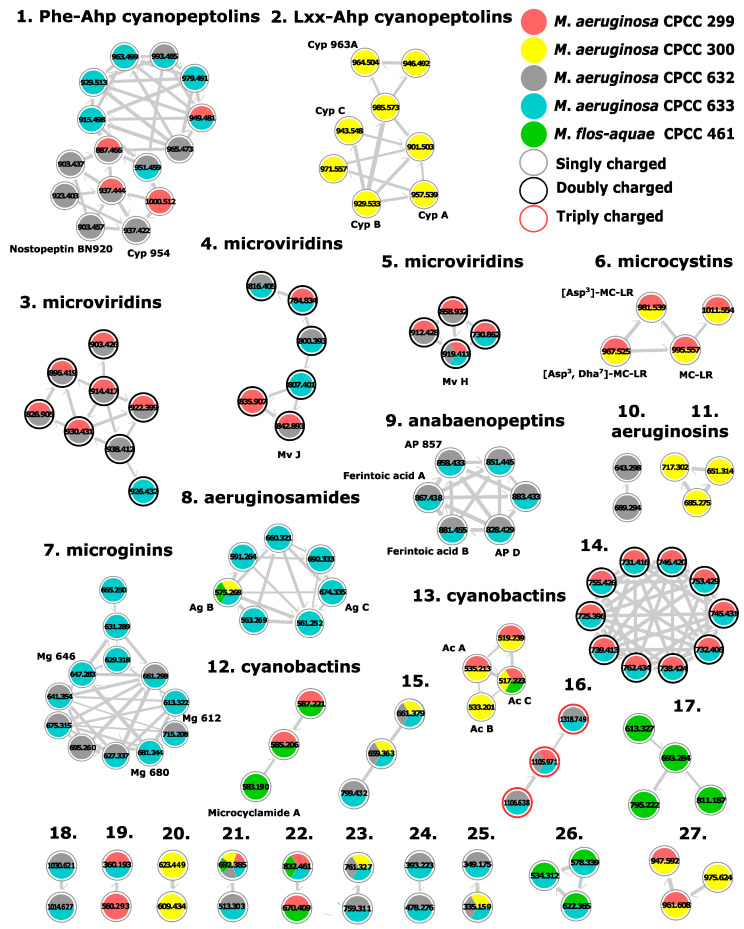
GNPS molecular network generated from cyanopeptide MS/MS spectra from the five studied *Microcystis* strains. Nodes are colour-coded pie charts labelled with precursor ion *m/z*. The width of lines connecting nodes is proportional to the corresponding cosine score. Precursor and product ion *m*/*z* tolerance were set to 0.02 and 0.03 Da, respectively. The minimum base peak intensity was set at 5 × 10^6^. Clustering parameters required a minimum cosine similarity score of 0.6 and five matching product ions. Abbreviations: Ac, aerucyclamide; Ag, aeruginosamide; AP, anabaenopeptin; Cyp, cyanopeptolin; MC, microcystin; Md, microcyclamide; Mg, microginin; Mv, microviridin.

Non-targeted metabolomics was used to determine the intracellular cyanopeptide diversity from five *Microcystis* strains (Figure 2 and Figure 3). Cells from triplicate cultures of each *Microcystis* strain grown in BG-11 medium were extracted with 80% aqueous methanol. The resulting extracts were analyzed by LC-MS/MS in positive electrospray ionization (ESI) mode using a data-dependent acquisition (DDA) experiment. A total of 262 features were detected with the *xcms* bioinformatics package in R after removing features attributed to the media. Principal component analysis (PCA) was used to visualize the variation between strains based on cyanopeptide *m/z* and their relative abundances (Figure 2; top). The PCA plot displayed four distinct groups, with *M. aeruginosa* CPCC 299 and CPCC 300 and *M. flos-aquae* CPCC 461 generating unique cyanopeptide profiles. *M. aeruginosa* CPCC 299 and CPCC 300 grouped closely together in the top left quadrant of the PCA plot, suggesting similarity in their intracellular cyanopeptide profiles. *M. aeruginosa* CPCC 632 and CPCC 633 overlapped in the PCA plot, indicating these two strains produce similar cyanopeptides. The cyanopeptide profile of *M. flos-aquae* CPCC 461 was different compared with the four *M. aeruginosa* strains, appearing alone in the bottom left quadrant of the PCA plot.

Factor loading was used to determine which cyanopeptide features contributed to the observed metabolome differences between the studied *Microcystis* strains (Figure 2). With the 262 features, a Kruskal-Wallis test for non-parametric data with Benjamini-Hochberg correction was performed to identify metabolites that statistically contributed to the cyanopeptide variation observed in the PCA plot (*p* < 0.05). The test detected 140 statistically different features that appear as red arrows (*p* < 0.05) in the factor loadings plot (Figure 2; bottom). The coordinate location of red arrows specifies cyanopeptides produced by each strain and aligns with the PCA plot. For example, the red arrows for MC-LR (*m*/*z* 995.5565 [M + H]^+^; feature 7) and [Asp^3^] MC-LR (*m*/*z* 981.5391 [M + H]^+^; feature 8) appear in the top left quadrant. These two microcystins were exclusively produced by *M. aeruginosa* CPCC 299 and 300, which also appear in the top left quadrant of the PCA plot. Further, the anabaenopeptins ferintoic acid A (*m*/*z* 867.4378 [M + H]^+^; feature 23) and ferintoic acid B (*m*/*z* 881.4552 [M + H]+; feature 24) appear in the right quadrants of the loadings plot and are exclusively produced by *M. aeruginosa* CPCC 632 and 633. Interpretation of the multivariate metabolomics data showed unique strain-specific cyanopeptide profiles from the studied strains (Figure 2 and Figure 3; Tables S3 and S4).

### 2.2. Molecular Networking with GNPS to Visualize Strain-Specific Cyanopeptide Diversity

GNPS molecular networking was also performed to better describe cyanopeptide diversity from the studied *Microcystis* strains. Cytoscape was used to visualize clusters of structurally related cyanopeptides with cosine scores above 0.6 and a minimum of five matching product ions. Each node represents a precursor ion feature and edge thickness signifies the magnitude of the cosine similarity score between respective nodes. To target cyanopeptides and deconvolute the network, features from blanks, features from outside the retention time range 2.0–6.5 min, features below 400 *m/z,* and clusters containing a single node were removed. The resulting molecular network for the five *Microcystis* strains shows 27 clusters, 126 nodes, and 232 edges (Figure 3). Nodes are colour-coded to show which strains produce each cyanopeptide. In agreement with the multivariate analysis, GNPS molecular networking revealed that each strain biosynthesized unique strain-specific cyanopeptide mixtures. Explicitly, the studied strains produced different mixtures of cyanopeptolins (clusters 1–2), microviridins (clusters 3–5), microcystins (cluster 6), microginins (cluster 7), cyanobactins (clusters 8, 12–13), anabaenopeptins (cluster 9), aeruginosins (clusters 10–11), and groups of related metabolites that could not be identified (clusters 15–27). In accordance with the multivariate analysis, molecular networking showed that *M. aeruginosa* CPCC 632 and 633 produced similar cyanopeptide profiles. Annotation of the molecular network was achieved by comparisons to analytical standards, interpretation of product ion patterns including identifying cyanopeptide diagnostic product ions generated from unique structural features, comparisons to the literature, and database searching. LC-HRMS data for all cyanopeptides and unidentified nodes in Figure 3 are tabulated in the Supplementary Information Table S4.

#### 2.2.1. Cyanopeptolins

**Figure 4:**
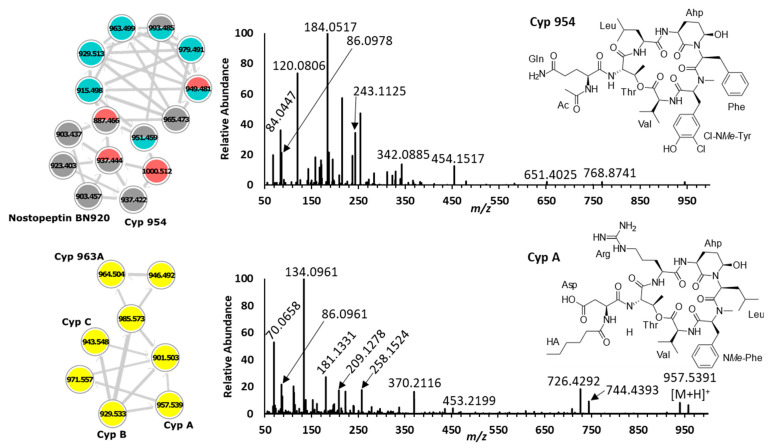
GNPS cluster 1 (**top left**) shows that *M. aeruginosa* 299, 632, and 633 produce different cyanopeptolin mixtures with the Phe–Ahp partial sequence. This partial sequence was identified by product ions at *m*/*z* 215.1175 [Phe–Ahp+H-H_2_O-CO]^+^ and *m*/*z* 243.1125 [Phe–Ahp+H-H_2_O]^+^ (**top right).** GNPS cluster 2 (**bottom left**) shows that *M. aeruginosa* CPCC 300 (yellow) was the only strain studied that produced cyanopeptolins with the Lxx–Ahp partial sequence. This partial sequence was determined by *m*/*z* 181.1331 [Lxx–Ahp+H-H_2_O-CO]^+^ and *m*/*z* 209.1278 [Lxx–Ahp+H-H_2_O]^+^ (**bottom right**).

Cyanopeptolins are non-ribosomal depsipeptides that possess a characteristic 3-amino-6-hydroxy-2-piperidone (Ahp) within their hexacyclic moiety [30]. They are generally known as protease inhibitors, and select congeners have shown toxicity towards *Caenorhabditis elegans, Danio rerio, Daphnia magna*, and *Thamnocephalus platyurus* in the low micromolar range [31,32,33]. Partial cyanopeptolin sequences were determined from diagnostic product ions. For example, Lxx–Ahp and Phe–Ahp cyanopeptolins are distinguished by product ions at *m*/*z* 181.1331 [Lxx–Ahp+H-H_2_O-CO]^+^ and *m*/*z* 215.1183 [Phe–Ahp+H-H_2_O-CO]^+^, respectively [28]. Additional sequence coverage can be determined by identifying the *N*Me-aromatic amino acid immonium ion present in all cyanopeptolins. Common aromatic residues include *N*Me-Phe (*m*/*z* 134.0963), *N*Me-Tyr (*m*/*z* 150.0912), and Cl-*N*Me-Tyr (*m*/*z* 184.0523). Different cyanopeptolin mixtures were produced by each of the four *M. aeruginosa* strains studied; however, none were detected from *M. flos-aquae* CPCC 461 (Figure 3 and Figure 4). Twenty-three cyanopeptolins were detected that comprise two clusters and several putative new compounds (Figure 3; clusters 1 and 2). Cluster 1 shows fifteen cyanopeptolins with the Phe–Ahp partial sequence from *M. aeruginosa* CPCC 299, 632, and 633. Cluster 2 shows eight cyanopeptolins with the Lxx–Ahp partial sequence exclusively from *M. aeruginosa* CPCC 300. Known cyanopeptolins from *M. aeruginosa* CPCC 300 include cyanopeptolins A-C and 963A, also known from *M. aeruginosa* PCC 7806 [30,34]. These two strains have similar cyanopeptide profiles, with overlapping production of cyanopeptolins, microcystins, and cyanobactins [30,34,35]. Cyanopeptolins are produced by several common bloom-forming species and some studies have documented their freshwater concentrations approaching effect levels [36,37].

#### 2.2.2. Microviridins

Microviridins are *N*-acyl trideca-(13 residues) and tetradeca-(14 residues) RiPP natural products [38,39]. Their unusual tricyclic scaffold results from intramolecular ω-ester and ω-amide bonds. Lactams are formed via the ω-carboxy groups of glutamate or aspartate with the ε-amino group of lysine and lactones through the esterification of glutamate or aspartate ω-carboxy moieties with the hydroxyl groups of serine and threonine. Whereas the microviridin central sequence motif where lactones and lactams are formed is generally conserved, their N- and C-termini are variable, resulting in most of the group’s structural diversity [40]. Unlike the other cyanopeptide groups investigated, microviridins are composed entirely of proteinaceous amino acids. Microviridins are also much larger (1600–1900 Da) than most *Microcystis* cyanopeptides [11]. As such, microviridins are readily detected by their doubly or triply charged ions and amino acid immonium ions in HRMS and MS/MS spectra, respectively. Eighteen unique microviridins were produced by *M. aeruginosa* CPCC 299, 632, and 633 (Figure 3; clusters 3–5). Microviridin H and J were identified based on molecular formula and the presence of correct amino acid immonium ions within their respective structures [38,41]. Immonium ions for exo-amino acids within the microviridin scaffold are hypothesized to distinguish the three microviridin clusters within the GNPS analysis. Microviridins are inhibitors of serine proteases and some variants are toxic to *Daphnia* by interfering with its molting process [41]. Structural activity relationships have connected potency with the identity of residue 5 [39]. For example, microviridins with Leu at position 5 (microviridins B, C, G, H,) are potent elastase inhibitors (IC_50_ 0.01–58 μM), whereas congeners with Met, Tyr, or Phe (microviridins D, E, F) at this position have little or no activity [38,39,41].

#### 2.2.3. Microcystins

The microcystins constitute more than three hundred potent hepatotoxins and possible human carcinogens (IARC Group 2B) [13]. Due to their toxic properties, microcystins are monitored in drinking and recreational waters in many jurisdictions. For example, the WHO has set provisional guideline values of 1 µg/L and 24 µg/L for microcystins in drinking and recreational waters, respectively [42]. Despite generally occurring as mixtures, these guidelines were derived using only microcystin-LR, one of the more potent and common congeners. Microcystins were identified by product ions at *m*/*z* 135.0808 and *m*/*z* 163.1118 in their MS/MS spectra, resulting from their characteristic β-amino acid Adda that is present within ~80% of known congeners, and *m*/*z* 213.0865 for Glu-Mdha [13,29]. *M. aeruginosa* CPCC 299 and CPCC 300 produced the same four microcystins including MC-LR and [Asp^3^]-MC-LR (Figure 3; cluster 6). These congeners are known from these strains [43,44]. Two additional microcystins (*m*/*z* 967.5254 [M + H]^+^ and 1011.5535 [M + H]^+^) were also detected. No product ion at *m*/*z* 213.0865 was observed in the MS/MS spectrum for *m*/*z* 967.5254 [M + H]^+^, suggesting it as [Asp^3^, Dha^7^]-MC-LR, which is structurally similar to the other confirmed congeners. The other microcystin could not be identified from the literature. Microcystins represent a small number of the total cyanopeptides detected in this study (Figure 3). This highlights the need to better understand the potential toxicity and environmental concentrations of non-microcystin cyanopeptides.

#### 2.2.4. Microginins

**Figure 5:**
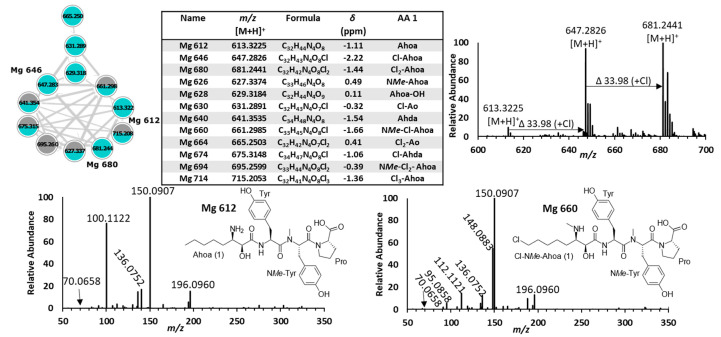
GNPS cluster 7 (**top left**) shows that *M. aeruginosa* CPCC 632 (grey) and 633 (blue) produce different microginin mixtures. LC-MS analysis (**top right**) of co-eluting of Mg 612 (Ahoa), 646 (Cl-Ahoa), and 680 (Cl_2_-Ahoa) with different N-termini residues. The structure of Mg 612 (**bottom left**) was determined by its MS/MS spectrum, which showed product ions at *m*/*z* 100.1122 (Ahoa), *m*/*z* 70.0658 (Pro), *m*/*z* 136.0752 (Tyr), and *m*/*z* 150.0910 (N*Me*Tyr). The structure of a related new variant, Mg 660 (**bottom right**), with a rare Cl-N*Me-*Ahoa moiety was identified by a product ion at *m*/*z* 148.0833 (Cl-N*Me-*Ahoa).

Microginins are linear cyanopeptides biosynthesized by hybrid NRPS–polyketide pathways [25]. They possess a characteristic N-terminal β-amino-α-hydroxy-decanoic or octanoic acid: 3-amino-2-hydroxydecanoic acid (Ahda) and 3-amino-2-hydroxyoctanoic acid (Ahoa) [45,46]. These moieties are variably chlorinated at their termini or *N*-methylated, which increases microginin structural diversity [10,47]. Most microginins possess four to six residues and are known enzyme inhibitors, specifically angiotensin-converting enzyme and aminopeptidases [45,48]. Microginins were identified by product ions resulting from their characteristic N-terminal Ahda and Ahoa groups at *m*/*z* 128.1433 and *m*/*z* 100.1122. The Ahoa product ion is isobaric with N*Me*-Lxx, which can confound their analysis. Increases in *m*/*z* 33.9606 from Ahda and Ahoa product ions were indicative of chlorination. For example, *m*/*z* 162.1039 and *m*/*z* 196.0645 are characteristic of mono- and di-chlorinated Ahda, respectively. Further, increases in *m*/*z* 14.0156 for these product ions were indicative of N-*Me* Ahda and Ahoa moieties. For example, *m*/*z* 114.1278 (N*Me*-Ahoa), *m*/*z* 148.0883 (N*Me*-Cl-Ahoa), and *m/z* 182.0494 (N*Me*-Cl_2_-Ahoa). Twelve microginins comprised of four residues were detected from *M. aeruginosa* CPCC 632 and 633 (Figure 3; cluster 7). Eleven microginins were produced by *M. aeruginosa* CPCC 633, and six were produced from *M. aeruginosa* CPCC 632. Interpretation of isotopic peak profiles, characteristic product ions, and immonium ions revealed the known microginins 613, 646, and 680 from *M. aeruginosa* CPCC 633 [46]. The remaining nine microginins represent new compounds where all structural changes between congeners arise from their N-terminal moiety given their identical Pro, Tyr, N*Me*Tyr immonium ions. Of which, seven were chlorinated, three were N*me*, two contained an Ahda scaffold, and, interestingly, two possess a putative unusual 3-amino-octanoic acid (Ao) moiety (Figure 5). Few studies have reported on microginin toxicity and environmental concentrations [6].

#### 2.2.5. Cyanobactins

Cyanobactins are a large group of cyclic and linear RiPP peptides that are formed by the proteolytic cleavage and cyclisation of precursor peptides [49,50,51]. Increased structural diversity for this group arises from the heterocyclisation and oxidation of amino acids and their posttranslational prenylation. Oxazoles and thiazoles resulting from the heterocyclisation and subsequent oxidation of Cys, Ser, and Thr residues are common cyanobactin structural features [11]. Interpretation of cyanobactin HRMS and MS/MS data was aided by the mass defect of sulfur atoms. Fourteen cyanobactins were detected from *M. aeruginosa* CPCC 299, 300, and 633 and *M. flos-aquae* CPCC 461 that constitute three clusters (Figure 3; clusters 8, 12, 13). *M. aeruginosa* CPCC 633 produced seven linear cyanobactins called aeruginosamides (Figure 3; cluster 8). This cyanobactin subgroup contain a C-terminal prenylated Phe residue, 1,1-dimethylallyl-Phe (Dma-Phe), and an N-terminal Tzl-O*Me* (Tzc). These characteristic features generate product ions at *m*/*z* 188.1428 [Dma–Phe-CO+H]^+^ and *m*/*z* 144.0109 (Tzc). Aeruginosamides B and C from *M. aeruginosa* CPCC 633 are also reported from *M. aeruginosa* PCC 9432 [49]. Aeruginosamide C was also detected from *M. aeruginosa* CPCC 300 and *M. flos-aquae* CPCC 461. The cyclic cyanobactin microcyclamide A and two related reduced compounds that contain an unusual N*Me*-histidinyl were produced by *M. flos-aquae* CPCC 461 (Figure 3; cluster 12) [52]. Based on interpretation of MS/MS patterns, structural differences are attributed to the reduction of thiazole to thiazoline moieties. The cyclic aerucyclamides A–C and a related compound were produced by *M. aeruginosa* CPCC 299 and 300 and *M. flos-aquae* CPCC 461 (Figure 3; cluster 13). Aerucyclamides were first reported from *M. aeruginosa* PCC 7806, which produces many of the same cyanopeptides as *M. aeruginosa* CPCC 300 [35,53]. Cyanobactins are common cyanopeptides, where between 10 to 30% of all cyanobacteria are estimated to possess a cyanobactin biosynthetic pathway [49]. They have received attention for their desirable properties including multidrug reversing, antiviral, antimalarial, and allelopathic activities and have been the focus of anticancer screening programs due to their cytotoxicity towards tumor cell lines [49]. Aerucyclamides have shown toxicity to fairy shrimp (*T. platyurus*; LC_50_ 30.5–33.8 μM) and microcyclamide has to zebra fish (*D. rerio*; LC_50_ 42.98 µg mL^−1^) [53]. It is unclear if cyanobactin toxicity is ecologically relevant as limited data exist and their environmental concentrations are largely unknown.

#### 2.2.6. Anabaenopeptins

**Figure 6:**
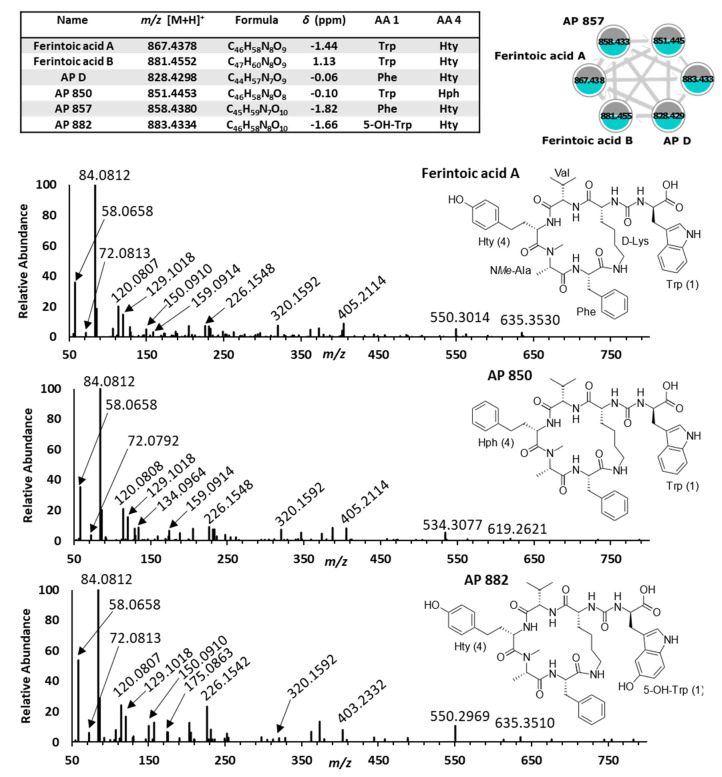
Tabulated HRMS data (**top left**) for GNPS cluster 9 (**top right**) shows *M. aeruginosa* CPCC 632 (grey) and 633 (blue) produce the same anabaenopeptin mixtures. Representative MS/MS spectra are shown for ferintoic acid A (**middle top**) and new structures AP 850 (**middle bottom**) and AP 882 (**bottom**). The molecular formula of anabaenopeptin 850 suggests a loss of an oxygen atom compared with ferintoic acid A. The MS/MS spectrum of anabaenopeptin 850 showed a product ion at *m*/*z* 134.0964 (Hph) instead of a Hty immonium ion accounting for the structural change. The molecular formula of anabaenopeptin 882 suggests the gain of an oxygen compared with ferintoic acid A. The MS/MS spectrum of anabaenopeptin 882 showed a product ion at *m*/*z* 175.0863 (5-OH-Trp) instead of Trp.

Anabaenopeptins are group of cyclic hexapeptides derived from NRPS pathways produced by numerous cyanobacteria genera [54]. Their structures contain a characteristic ureido linkage connecting a D-lysine residue within a five-residue ring to an exocyclic amino acid [11,55]. The cyclic moiety is comprised of variable proteinogenic or non-proteinogenic residues that are often N*Me*-residues [56]. Anabaenopeptins were identified by an intense product ion at *m*/*z* 84.0812 and a complementary peak at *m*/*z* 129.1018, which are indicative of lysine present within all congeners. *M. aeruginosa* CPCC 632 and 633 produced the same six anabaenopeptins (Figure 6), where ferintoic acid A was confirmed with a reference material. All anabaenopeptins contained an N*Me*-Ala residue and have been previously documented in the environment [22,57]. Anabaenopeptins are serine protease and phosphatase inhibitors; chymotrypsin, carboxypeptidase A (CPA), elastase, trypsin, and protein phosphatase 1 are inhibited in the low μM range [11]. Enzyme inhibition likely explains their toxicity towards zooplankton, crustaceans, and the model organism *C. elegans* [32]. Although their toxicity is lower than microcystins, synergistic inhibitory effects have been observed between the cyanopeptide groups [58]. Further, anabaenopeptins have been shown to dominate in surface waters with blooms [59].

#### 2.2.7. Aeruginosins and Unknown Clusters

Aeruginosins are a group of linear NRPS tetrapeptides notably produced by *Microcystis* and *Planktothrix* [60]. Their structures are characterized by an N-terminal 4-hydroxyphenyllactic acid (Hpla), a 2-carboxy-6-hydroxyoctahydroindole (Choi) moiety and a C-terminal arginine derivative such as agmatine, argininol, or argininal [25]. Structural variability of these serine protease inhibitors is increased by chlorination and sulfation of the Choi and Hpla functionalities [11,61]. Aeruginosins were confirmed by diagnostic product ions at *m*/*z* 140.1064 and 122.0962 corresponding to Choi. *M. aeruginosa* CPCC 300 and CPCC 632 produced distinct sets of aeruginosins (Figure 3; clusters 10 and 11). Two aeruginosins that contain a phenylalanine and sulfated Choi group were produced by *M. aeruginosa* CPCC 632. From *M. aeruginosa* CPCC 300, three aeruginosins that contain a tyrosine were detected. None of the congeners within the molecular network matched the literature and three of five putative new structures contained a chlorinated Hpla residue.

Most cyanopeptides detected from the five *Microcystis* strains could not be confirmed with reference materials and represent new structures. Despite this, the interpretation HRMS and MS/MS data for cyanopeptides from groups with characteristic structural features was useful in deciphering chemical diversity for the five *Microcystis* strains investigated. Clusters 14 to 27 in Figure 3 represent groups of cyanobacteria metabolites that could not be identified. For example, cluster 14 shows ten metabolites produced by *M. aeruginosa* CPCC 299 and CPCC 633 that generated double charged ions in the *m*/*z* 720–770 [M + 2H]^2+^ range. These putative new cyanopeptides had amino acid immonium ions or related ions, *m*/*z* 70.0657 (Pro), *m*/*z* 86.0968 (Lxx), *m*/*z* 102.0553 (Glu), *m*/*z* 136.0755 (Tyr), and a diagnostic product ion *m*/*z* 115.0865 (C_5_H_11_N_2_O^+^) in their MS/MS spectra. This group of compounds will be the focus of future investigations. Further, none of the studied strains produced the cytotoxic aeruginoguanidines, based on interpreting datasets acquired in both positive and negative electrospray ionization modes.

### 2.3. Temporal Co-Production of Cyanopeptides

**Figure 7:**
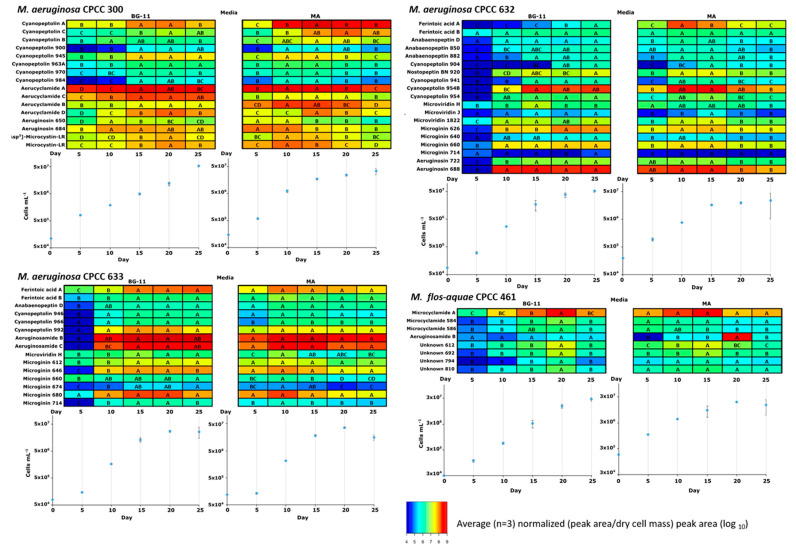
Heatmaps and growth curves (cells mL^−1^) showing relative cyanopeptide amounts produced by *M. aeruginosa* CPCC 300 (**top left**), 632 (**top right**), and 633 **(bottom left**) and *M. flos-aquae* CPCC 461 (**bottom right**) over a 25-day growth period in BG-11 and MA media. The heatmaps show the average normalized (peak area/dry cell mass) peak area (log_10_) for cyanopeptides identified in the GNPS analysis that maintained an HRMS peak area above 5 × 10^4^. A Tukey’s LSD test (*p* < 0.005) distinguished relative differences in cyanopeptide production at each interval (designated with letters).

The co-production dynamics of the dominant cyanopeptides identified by factor and GNPS analysis from *M. aeruginosa* CPCC 300, 632, and 633 and *M. flos-aquae* CPCC 461 were explored. Limited studies have examined the dynamics of non-microcystin cyanopeptide production, which is hindered by a lack of reference materials [62]. Sampled every 5 days for a 25-day period, cyanopeptides were included in this analysis if they maintained a peak area greater than 5 × 10^4^ at every time point. Whereas both BG-11 and MA medium are used for culturing *Microcystis*, MA has a higher pH (8.6), a bicine buffer, and higher amounts of trace elements with the exceptions of copper, which it omits, and iron. Orthophosphate, as K_2_HPO_4_ for BG-11, is replaced with Na_2_ β-glycerophosphate heptahydrate in MA [63]. The relative abundance of each cyanopeptide was normalized by dividing its peak area by collected dry cell mass, and a Tukey’s LSD test (*p* < 0.05) was used to distinguish statistically significant differences in the production of each cyanopeptide over time (Figure 7). Each of the four strains reached the stationary phase faster in MA than in BG-11 medium. For most cyanopeptides, an increase in relative abundance was observed in the mid-exponential phase followed by a slight decrease (Figure 7). This trend was harder to discern from the BG-11 data, where the strains grew slower, and for compounds with large peak areas. For example, cyanobactins (aerucyclamides A–D) produced by *M. aeruginosa* CPCC 300 appear to have higher intracellular amounts than other cyanopeptide groups at all growth stages. Without a standard, we could not quantify these cyanopeptides; however, this observation is in accordance with Natumi and Janssen, 2020 who examined cyanopeptide production for *M. aeruginosa* PCC7806 in WC medium. Similarly, microcyclamide A and the linear cyanobactins, aeruginosamides appear to be the dominant metabolites produced by *M. flos-aquae* CPCC 461 and *M. aeruginosa* CPCC 633, respectively. The qualitative cyanopeptide profiles for each of the strains was unchanged over the growth period in the two media. For microcystins, peak production during mid-exponential growth accompanied by an unchanged profile of congeners over the growth cycle have been reported in *Microcystis* [64,65]. Constitutive production of microcystins and microginins, where amounts did not change over the growth cycle, have also been observed in *Microcystis* [66]. Despite cyanopeptide groups being biosynthesized by independent pathways, similar relative amounts of cyanopeptide groups were maintained over the growth period. These data are in accordance with Natumi and Janssen, 2020 who hypothesized that synchronous cyanopeptide production is not related to requirements that change during the growth cycle. Some cyanopeptides are toxic towards grazing zooplankton, suggesting they have important ecological function [67]. However, the synchronous constitutive production of cyanopeptides indicates the potential for other important biological functions that remain to be elucidated. Harvesting *Microcystis* cells in the mid to late exponential phase will provide biomass with relatively high cyanopeptide amounts for the preparation of reference materials enabling future studies of their environmental distributions and biological activities.

## 3. Conclusions

Common bloom-forming cyanobacteria, including *Microcystis* species, produce incredibly diverse mixtures of cyanopeptides. Little is known about the environmental concentrations and biological functions of cyanopeptides released by blooms into freshwater ecosystems. In this study, we used mass-spectrometry-based metabolomics to determine the cyanopeptide profiles of five *Microcystis* strains and investigate their production dynamics over their growth cycle. Factor analysis and GNPS molecular networking revealed that each *Microcystis* strain produced a unique cyanopeptide profile. For each strain, peak cyanopeptide production was observed during mid-exponential growth. Further, the identity of the cyanopeptides produced by each strain remained unchanged over their growth cycle. The results of this study will guide the cultivation of *Microcystis* strains for analytical standard preparation to facilitate exposure and bioactivity assessments for common and abundant non-microcystin cyanopeptides detected from blooms. The synchronous constitutive production of multiple cyanopeptide groups by *Microcystis* highlights the need to better understand the environmental concentrations and biological functions for chemically diverse cyanopeptides.

## 4. Materials and Methods

### 4.1. Growth of Microcystis Strains

Four *Microcystis aeruginosa* strains (CPCC 299, CPCC 300, CPCC 632, CPCC 633) and *M. flos-aquae* CPCC 461 were obtained from the Canadian Phycological Culture Centre (CPCC) at the University of Waterloo, Waterloo, ON, Canada. Information on the origin of each strain can be found in the Supplemental Data (Table S1). Triplicate cultures of each strain were grown in 250 mL Erlenmeyer flasks (Pyrex; Stoke on Trent, UK) that contained 50 mL of BG-11 or MA media. Cultures were maintained in a growth chamber (Conviron E15; Winnipeg, MB, Canada) under cool white fluorescent light (30 μE m^−2^ s^−1^) using a light/dark regime of 12 h at 25 °C. Flasks were swirled every 2 days and late-exponential cells were harvested after 20 days for factor and GNPS molecular networking analysis. To monitor culture growth, *Microcystis* cells were counted using a Hausser Scientific Co. (Horsham, PA, USA) counting chamber (catalogue #3200) and glass micro cover (22 × 22 mm; VWR; Radnor, PA, USA) under a VWR VistaVision Microscope.

### 4.2. Cyanopeptide Extraction from Microcystis Cells

To extract intracellular cyanopeptides, a 25 mL aliquot of *Microcystis* cells grown in BG-11 medium were harvested on glass microfiber filters (pre-weighed and dried overnight at 60 °C; Whatman, GF/A, diameter 47 mm, ~1.6 µm, Maidstone, UK) using a Millipore (Burlington, MA, USA) filtration apparatus. After drying in an oven at 60 °C overnight, the glass microfiber filters with *Microcystis* cells were transferred to disposable glass culture tubes (16 × 100 mm, VWR) and 14 mL of 80% aqueous methanol was added. The tubes were vortexed and sonicated for 30 s each and stored at −20 °C for 1 h. Tubes were subsequently thawed to room temperature and the freeze–thaw cycle, including vortexing and sonication, was repeated two additional times. The resulting methanolic extracts containing intracellular cyanopeptides were filtered through 0.22 μm PTFE syringe filters (ChromSpec, Inc., Brockville, ON, Canada) into amber vials and dried under a gentle stream of nitrogen gas. Extracts of *Microcystis* cells were then reconstituted in HPLC grade methanol, transferred to 2 mL amber HPLC vials (Agilent, Santa Clara, CA, USA), dried under a gentle stream of nitrogen gas, and stored at −20 °C prior to LC-MS/MS analysis.

### 4.3. Acquisition of Non-Targeted LC-MS/MS Data

Prior to analysis, the dried residues were reconstituted in 1 mL of 90% aqueous methanol and vortexed for 15 s. Non-targeted LC-MS/MS datasets of *Microcystis* cyanopeptides were acquired using an Agilent 1290 HPLC (Agilent Technologies, Santa Clara, CA, USA) coupled to a Q-Exactive Orbitrap (Thermo Fisher Scientific, Waltham, MA, USA) mass spectrometer [28]. Chromatography conditions included an Eclipse Plus C18 RRHD column (2.1 × 100 mm, 1.8 μm; Agilent Technologies) maintained at 35 °C and a mobile phase consisting of 0.1% formic acid (mobile phase A) and acetonitrile with 0.1% formic acid in water (mobile phase B). The 10.5 min gradient program optimized for cyanopeptide analysis increased from 0 to 35% B over 1.5 min, increased again to 45% B over the following 2.5 min, and finally increased to 100% B over 2 min, where it was held at 100% B for 3 min prior to returning to 0% B over 0.5 min and maintained at 0% B for 1 min. Five µL of each extract was injected and resolved at a flow rate of 0.3 mL/min.

Heated electrospray ionization in positive mode was used with the following settings: capillary voltage, 3.9 kV; capillary temperature, 400 °C; sheath gas, 19 units; auxiliary gas, 8 units; probe heater temperature, 450 °C; S-Lens RF level, 45.00. MS data were acquired using data-dependent acquisition (DDA) experiments that included a full MS scan at 70,000 resolution (a scan range of 106.7–1600 *m*/*z*), automatic gain control target 3 × 10^6^, and a maximum injection time of 250 ms. The five highest intensity ions were selected from each full scan for MS/MS analysis using a 1.0 Da isolation window and were analyzed using the following conditions: resolution, 17,500; automatic gain control target, 1 × 10^6^; max IT, 64 ms; normalized collision energy, 37.5; intensity threshold, 1.5 × 10^5^; dynamic exclusion, 5 s.

### 4.4. Processing of Non-Targeted LC-MS/MS Datasets

#### 4.4.1. PCA and Factor Loading in R

Raw MS data files acquired in positive electrospray ionization mode for the extracts of triplicate cultures of each *Microcystis* strain were converted to centroid mzML files with MSConvert v3.1.19 (http://proteowizard.sourceforge.net/, accessed on 21 March 2023) with peak picking set at MS level 1 [68]. The mzML files were subsequently processed in this file format in R 4.1.3 (https://www.r-project.org/, accessed on 21 March 2023). Feature peak areas were extracted using the *xcms* package; the parameters are in Supplementary Information Table S2. All zero values were imputed with two-thirds of the lowest peak area value measured per metabolite [69]. Features corresponding to cyanopeptide isotopic peaks and redundant ESI adducts were removed manually. Deconvolution of the extracted peak list was achieved with the removal of features outside the retention time range of 2–6.5 min, below *m*/*z* 400, and detected in the media blanks. Remaining feature peak areas were log_10_ transformed and Pareto scaled. Within R, PCA and factor loadings were performed using the packages *MetabolAnalyze* and *FactoMineR* [70,71]. All dimensions of the PCA were analyzed; however, the first and fourth dimensions were plotted against each other as they explained the most variation between the strains studied (Figure 2). The HRMS data from triplicate *Microcystis* cultures were grouped and a Kruskal-Wallis test for non-parametric data with a Benjamini–Hochberg correction was performed in R to determine the statistical significance of the features and their contribution to the PCA [72]. Features with a *p*-value < 0.05 were deemed significant between strains (groups) and labelled red within the factor loadings plot (Figure 2). The factor loadings and PCA data were exported to excel and the identified cyanopeptides were numbered. Cyanopeptides were identified by comparison of *m/z* and RT with available analytical standards or putatively by the interpretation of MS/MS spectra, literature surveys, and database searching: GNPS and CyanoMetDB [10]. The reference materials used for confirmatory purposes were MC-LR (NRC), [Dha^7^] MC-LR (NRC), and ferintoic acid A (Enzo Life Sciences). Cyanopeptides identified by HRMS analysis are tabulated in Supplementary Information Tables S3 and S4. Representative LC-HRMS spectra for studied stains are also found in the Supplementary Information Figures S1–S5.

#### 4.4.2. GNPS Molecular Networking

For molecular networking in GNPS, the raw HRMS data files were converted to mzML files with MSConvert with peak picking set at MS level 1–2. The mzML files for the intracellular extracts of each *Microcystis* strain and a media blank were uploaded to the GNPS network (https://gnps.ucsd.edu/ProteoSAFe/static/gnps-splash.jsp, accessed on 21 March 2023) with FileZilla (https://filezilla-project.org/, accessed on 21 March 2023) through the ccms host. Files were analyzed with the following parameters: precursor ion mass tolerance, 0.02 *m*/*z*; fragment ion mass tolerance, 0.03 *m*/*z*; min pairs cosine score, 0.6; Network TopK, 10; maximum connected component size, 100; minimum matched fragment ions, 5; minimum cluster size, 2; and MSCluster, on. The graphML file format from the GNPS website was uploaded into Cytoscape for visualization (https://cytoscape.org/, accessed on 21 March 2023). The blank data file was used for data deconvolution with all metabolites present in the blank deleted from the network. Further deconvolution included the elimination of metabolites outside the retention time range 2–6.5 min, below *m*/*z* 400, and any remaining single nodes. Nodes were coloured based on which data file (*Microcystis* strain) they were present in and outlined to describe their charge state. Normalized peak area data for dominant cyanopeptides included in the GNPS analysis are visualized in Figure S6.

#### 4.4.3. Heat Map Analysis to Illustrate Temporal Cyanopeptide Co-Production

Cyanopeptides identified from the molecular networking analysis were selected for heat map analysis to examine temporal cyanopeptide co-production and the influence of two defined media (BG-11 and MA [63]). *M. aeruginosa* CPCC 300, CPCC 632, and CPCC 633 and *M. flos-aquae* CPCC 461 were grown in triplicate batch cultures and sampled every five days: days 5, 10, 15, 20, and 25. The growth of strains, generation of intracellular extracts, and HRMS analysis were performed as described above (4.1–4.3). Peak areas for selected cyanopeptides were normalized with dried cyanobacteria biomass. Cyanopeptides were excluded if their peak areas did not exceed 5 × 10^4^ at any time point. Heat maps were generated with log_10_ normalized peak area per strain, per media in R using *gplots* for heat map generation and *ColorRamps* for visualization. Peak area data and dry cyanobacteria cell mass amounts can be found in the Supplementary Information.

## Data Availability

Additional data will be made available upon request.

## Supplementary Materials

The following supporting information can be downloaded at: https://www.mdpi.com/article/10.3390/toxins15040254/s1, Table S1: Collection and specific growth rate information for studied *Microcystis* strains in BG-11; Table S2: The peak picking parameters used with the bioinformatics package xcms; Table S3: Cyanopeptide groups produced by *M. aeruginosa* CPCC 299, 300, 632, and 633 and *M. flos-aquae* CPCC 461; Table S4: HRMS data for cyanopeptide produced by *M. aeruginosa* CPCC 299, 300, 632, and 633 and *M. flos-aquae* CPCC 461 identified in the GNPS analysis. Figures S1–S5: LC-HRMS spectra for *Microcystis* intracellular extracts; Figure S6: Normalized relative abundance (peak area/dry cell mass) of dominant cyanopeptides produced by the five studied *Microcystis* strains included in the GNPS analysis. Peak area and dry cell mass data used to generate Figure 7 are found in: Supplementary Information_heatmap data_tabulated.xls. Refs [73,74] are cited in Supplementary Materials.
