# Supplementary material for: Metabolomics Reveals Strain-Specific Cyanopeptide Profiles and Their Production Dynamics in Microcystis aeruginosa and M. flos-aquae
Source: Toxins (Basel). 2023 Mar 31;15(4):254. doi: 10.3390/toxins15040254 (PMC10147050; doi:10.3390/toxins15040254)
Supplement: Supplementary file 1 [file toxins-15-00254-s001.zip › toxins-2280541 Supplementary Table S1-S3 Figure S1-S6.pdf]

toxins MDPI

**Table S1.** Collection and specific growth rate information for studied *Microcystis* strains in BG-11.

| Strain | Collection Data | |
|---|---|---|
| | Date | Location |
| *M. aeruginosa* CPCC 299 | 08/90 | Pretzlaff Pond, AB, Canada |
| *M. aeruginosa* CPCC 300 | 08/90 | Pretzlaff Pond, AB, Canada |
| *M. flos-aquae* CPCC 461 | 08/75 | Fox River, WI, USA |
| *M. aeruginosa* CPCC 632 | 10/48 | Lake Mendota, WI, USA |
| *M. aeruginosa* CPCC 633 | 10/54 | Little Rideau Lake, ON, Canada |

**Table S2.** The peak picking parameters used with the bioinformatics package *xcms*.

| Parameter | Polarity | Scan Pre-Filter * | *m/z* Deviation (ppm) | s/n Ratio Cutoff | Peak width Range (secs) | Noise Level |
|---|---|---|---|---|---|---|
| **Set point** | + | 5 with minimum intensity of 5000 | 1 | 5 | 12–15 | 500,000 |

* Number of scans a metabolite must be present in with a minimum intensity of 5000.

**Table S3.** Cyanopeptide groups produced by *M. aeruginosa* CPCC 299, 300, 632 and 633, and *M. flos-aquae* CPCC 461.

| Cyanopeptide Group | *Microcystis* Strain (CPCC) | | | | |
|---|---|---|---|---|---|
| | 299 | 300 | 632 | 633 | 461 |
| Microcystin | + | + | | | |
| Anabaenopeptin | | | + | + | |
| Microginin | | | + | + | |
| Cyanopeptolin | + | + | + | + | |
| Cyanobactin | + | + | | + | + |
| Aeruginosin | | + | + | | |
| Microviridin | + | | + | + | |

**Table S4.** HRMS data for cyanopeptide produced by *M. aeruginosa* CPCC 299, 300, 632 and 633, and *M. flos-aquae* CPCC 461 identified in the GNPS analysis.

| Cluster # | Cyanotoxin group | Name | *m*/*z* | Formula | δ (ppm) | Rt (min) | *Microcystis* CPCC strain | | | | | Reference |
|---|---|---|---|---|---|---|---|---|---|---|---|---|
| | | | | | | | 299 | 300 | 461 | 632 | 633 | |
| 1 | Cyanopeptolin | Cyp 904 | 887.4652 $[M+H-H_2O]^+$ | $C_{46}H_{64}N_8O_{11}$ | −1.08 | 4.20 | + | | | + | | This study |
| 1 | Cyanopeptolin | Cyp 920 | 903.4367 $[M+H-H_2O]^+$ | $C_{43}H_{65}N_8O_{12}Cl$ | −1.37 | 3.40 | | | | + | | This study |
| 1 | Cyanopeptolin | NP BN920 | 903.4587 $[M+H-H_2O]^+$ | $C_{46}H_{64}N_8O_{12}$ | 0.10 | 3.25 | | | | + | | [73] |
| 1 | Cyanopeptolin | Cyp 932 | 915.4972 $[M+H-H_2O]^+$ | $C_{48}H_{68}N_8O_{11}$ | −0.29 | 4.85 | | | | | + | This study |
| 1 | Cyanopeptolin | Cyp 940 | 923.4039 $[M+H-H_2O]^+$ | $C_{45}H_{65}N_8O_{12}Cl$ | −0.45 | 3.38 | | | | + | | This study |
| 1 | Cyanopeptolin | Cyp 946 | 929.5116 $[M+H-H_2O]^+$ | $C_{49}H_{70}N_8O_{11}$ | −1.63 | 5.19 | | | | | + | This study |
| 1 | Cyanopeptolin | Cyp 954 | 937.4217 $[M+H-H_2O]^+$ | $C_{46}H_{63}N_8O_{12}Cl$ | −0.43 | 3.51 | | | | + | | [73] |
| 1 | Cyanopeptolin | Cyp 954B | 937.4441 $[M+H-H_2O]^+$ | $C_{49}H_{62}N_8O_{12}$ | −1.41 | 4.16 | + | | | + | | This study |
| 1 | Cyanopeptolin | Cyp 966 | 949.4821 $[M+H-H_2O]^+$ | $C_{51}H_{66}N_8O_{11}$ | 0.29 | 5.00 | + | | | | + | This study |
| 1 | Cyanopeptolin | Cyp 968 | 951.4596 $[M+H-H_2O]^+$ | $C_{50}H_{64}N_8O_{12}$ | −1.55 | 3.96 | | | | + | + | This study |
| 1 | Cyanopeptolin | Cyp 980 | 963.4964 $[M+H-H_2O]^+$ | $C_{52}H_{68}N_8O_{11}$ | −1.04 | 5.31 | | | | | + | This study |
| 1 | Cyanopeptolin | Cyp 982 | 965.4525 $[M+H-H_2O]^+$ | $C_{48}H_{67}N_8O_{12}Cl$ | 1.19 | 4.02 | | | | + | | This study |
| 1 | Cyanopeptolin | Cyp 992 | 979.4930 $[M+H-H_2O]^+$ | $C_{52}H_{68}N_8O_{12}$ | 0.73 | 4.79 | | | | | + | This study |
| 1 | Cyanopeptolin | Cyp 1010 | 993.4856 $[M+H-H_2O]^+$ | $C_{50}H_{71}N_8O_{12}Cl$ | 0.08 | 4.97 | | | | + | + | This study |
| 1 | Cyanopeptolin | Cyp 999 | 1000.5120 $[M+H]^+$ | $C_{51}H_{69}N_9O_{12}$ | −1.34 | 3.90 | + | | | | | This study |
| 2 | Cyanopeptolin | Cyp 900 | 901.5031 $[M+H]^+$ | $C_{44}H_{68}N_8O_{12}$ | 0.17 | 3.17 | | + | | | | This study |
| 2 | Cyanopeptolin | Cyp B | 929.5327 $[M+H]^+$ | $C_{46}H_{72}N_8O_{12}$ | −1.66 | 3.69 | | + | | | | [30] |
| 2 | Cyanopeptolin | Cyp C | 943.5481 $[M+H]^+$ | $C_{47}H_{74}N_8O_{12}$ | −1.90 | 3.70 | | + | | | | [30] |
| 2 | Cyanopeptolin | Cyp 945 | 946.4917 $[M+H]^+$ | $C_{49}H_{67}N_7O_{12}$ | −0.36 | 5.35 | | + | | | | This study |
| 2 | Cyanopeptolin | Cyp A | 957.5391 $[M+H]^+$ | $C_{46}H_{72}N_{10}O_{12}$ | −1.35 | 3.75 | | + | | | | [30] |
| 2 | Cyanopeptolin | Cyp 963A | 964.5040 $[M+H]^+$ | $C_{49}H_{69}N_7O_{13}$ | 1.53 | 5.31 | | + | | | | [34] |
| 2 | Cyanopeptolin | Cyp 970 | 971.5565 $[M+H]^+$ | $C_{47}H_{74}N_{10}O_{12}$ | 0.46 | 4.14 | | + | | | | This study |
| 2 | Cyanopeptolin | Cyp 984 | 985.5725 $[M+H]^+$ | $C_{48}H_{76}N_{10}O_{12}$ | 0.61 | 4.72 | | + | | | | This study |

| | | | | | | | | | | | | |
|---|---|---|---|---|---|---|---|---|---|---|---|---|
| 3 | Microviridin | Mv 1630 | 816.4052 [M+2H]$^{2+}$ | $C_{72}H_{112}N_{17}O_{26}$ | −0.35 | 2.81 | | | | + | + | This study |
| 3 | Microviridin | Mv 1651 | 826.9047 [M+2H]$^{2+}$ | $C_{86}H_{107}N_{16}O_{18}$ | −0.02 | 2.70 | + | | | + | | This study |
| 3 | Microviridin | Mv 1669 | 835.9076 [M+2H]$^{2+}$ | $C_{80}H_{113}N_{14}O_{25}$ | 0.36 | 2.79 | + | | | | | This study |
| 3 | Microviridin | Mv 1804 | 903.4258 [M+2H]$^{2+}$ | $C_{88}H_{114}N_{19}O_{23}$ | 2.00 | 2.80 | + | | | + | | This study |
| 3 | Microviridin | Mv 1856 | 922.3999 [M+2H]$^{2+}$ | $C_{87}H_{112}N_{17}O_{28}$ | −0.55 | 2.81 | + | | | + | | This study |
| 3 | Microviridin | Mv 1850 | 926.4323 [M+2H]$^{2+}$ | $C_{93}H_{116}N_{19}O_{22}$ | −2.18 | 2.86 | | | | | + | This study |
| 3 | Microviridin | Mv 1858 | 930.4302 [M+2H]$^{2+}$ | $C_{91}H_{116}N_{19}O_{24}$ | 1.13 | 2.87 | + | | | + | | This study |
| 3 | Microviridin | Mv 1874 | 938.4132 [M+2H]$^{2+}$ | $C_{88}H_{116}N_{17}O_{29}$ | −1.75 | 2.89 | | | | + | | This study |
| 4 | Microviridin | Mv 1567 | 784.8342 [M+2H]$^{2+}$ | $C_{80}H_{91}N_{14}O_{20}$ | 0.31 | 3.21 | + | | | | + | This study |
| 4 | Microviridin | Mv 1589 | 800.3928 [M+2H]$^{2+}$ | $C_{71}H_{109}N_{17}O_{25}$ | 0.61 | 2.64 | | | | + | + | This study |
| 4 | Microviridin | Mv J | 842.8925 [M+2H]$^{2+}$ | $C_{80}H_{105}N_{19}O_{22}$ | 1.31 | 2.75 | + | | | + | | [41] |
| 4 | Microviridin | Mv 1715 | 858.9321 [M+2H]$^{2+}$ | $C_{88}H_{115}N_{16}O_{20}$ | 1.36 | 2.90 | + | | | + | | This study |
| 4 | Microviridin | Mv 1822 | 912.4276 [M+2H]$^{2+}$ | $C_{88}H_{116}N_{19}O_{24}$ | −1.87 | 2.80 | + | | | + | | This study |
| 4 | Microviridin | Mv H | 919.4114 [M+2H]$^{2+}$ | $C_{89}H_{114}N_{17}O_{26}$ | −2.11 | 2.87 | + | | | + | + | [74] |
| 5 | Microviridin | Mv 1459 | 730.8624 [M+2H]$^{2+}$ | $C_{82}H_{91}N_{16}O_{10}$ | −0.15 | 2.44 | + | | | | + | This study |
| 5 | Microviridin | Mv 1612 | 807.4003 [M+2H]$^{2+}$ | $C_{72}H_{111}N_{17}O_{25}$ | 0.06 | 2.80 | | | | | + | This study |
| 5 | Microviridin | Mv 1790 | 896.4191 [M+2H]$^{2+}$ | $C_{80}H_{116}N_{19}O_{28}$ | 0.06 | 2.64 | + | | | + | | This study |
| 5 | Microviridin | Mv 1826 | 914.4168 [M+2H]$^{2+}$ | $C_{90}H_{112}N_{19}O_{23}$ | 0.66 | 2.81 | + | | | + | | This study |
| 6 | Microcystin | [Asp$^{3}$, Dha$^{7}$]-MC-LR | 967.5254 [M+H]$^{+}$ | $C_{47}H_{70}N_{10}O_{12}$ | −1.69 | 3.29 | + | + | | | | [13] |
| 6 | Microcystin | [Asp$^{3}$]-MC-LR | 981.5385 [M+H]$^{+}$ | $C_{48}H_{72}N_{10}O_{12}$ | −0.71 | 3.40 | + | + | | | | [43] |
| 6 | Microcystin | MC-LR | 995.5565 [M+H]$^{+}$ | $C_{49}H_{74}N_{10}O_{12}$ | 1.76 | 3.49 | + | + | | | | [43] |
| 6 | Microcystin | MC-1010 | 1011.5535 [M+H]$^{+}$ | $C_{49}H_{74}N_{10}O_{13}$ | −0.41 | 3.37 | + | + | | | | |
| 7 | Microginin | Mg 612 | 613.3225 [M+H]$^{+}$ | $C_{32}H_{44}N_{4}O_{8}$ | −1.11 | 2.53 | | | | | + | [46] |
| 7 | Microginin | Mg 626 | 627.3374 [M+H]$^{+}$ | $C_{33}H_{46}N_{4}O_{8}$ | 0.49 | 2.70 | | | | + | + | This study |
| 7 | Microginin | Mg 628 | 629.3184 [M+H]$^{+}$ | $C_{32}H_{44}N_{4}O_{9}$ | 0.11 | 2.31 | | | | | + | This study |
| 7 | Microginin | Mg 630 | 631.2891 [M+H]$^{+}$ | $C_{32}H_{43}N_{4}O_{7}Cl$ | −0.32 | 2.60 | | | | | + | This study |

| | | | | | | | | | | | | |
|---|---|---|---|---|---|---|---|---|---|---|---|---|
| 7 | Microginin | Mg 640 | 641.3535 [M+H]+ | $C_{34}H_{48}N_4O_8$ | −1.54 | 2.75 | | | | + | + | This study |
| 7 | Microginin | Mg 646 | 647.2826 [M+H]+ | $C_{32}H_{43}N_4O_8Cl$ | −2.22 | 2.56 | | | | | + | [46] |
| 7 | Microginin | Mg 660 | 661.2985 [M+H]+ | $C_{33}H_{45}N_4O_8Cl$ | −1.66 | 2.70 | | | | + | + | This study |
| 7 | Microginin | Mg 664 | 665.2503 [M+H]+ | $C_{32}H_{42}N_4O_7Cl_2$ | 0.41 | 3.04 | | | | | + | This study |
| 7 | Microginin | Mg 674 | 675.3148 [M+H]+ | $C_{34}H_{47}N_4O_8Cl$ | −1.06 | 2.72 | | | | + | + | This study |
| 7 | Microginin | Mg 680 | 681.2441 [M+H]+ | $C_{32}H_{42}N_4O_8Cl_2$ | −1.44 | 2.65 | | | | | + | [46] |
| 7 | Microginin | Mg 694 | 695.2599 [M+H]+ | $C_{33}H_{44}N_4O_8Cl_2$ | −0.39 | 2.89 | | | | | + | This study |
| 7 | Microginin | Mg 714 | 715.2053 [M+H]+ | $C_{32}H_{41}N_4O_8Cl_3$ | −1.36 | 2.86 | | | | + | + | This study |
| 8 | Aeruginosamide | Ag 560 | 561.2522 [M+H]+ | $C_{31}H_{36}N_4O_4S$ | −0.68 | 2.97 | | | | | + | This study |
| 8 | Aeruginosamide | Ag 562 | 563.2695 [M+H]+ | $C_{31}H_{38}N_4O_4S$ | −1.86 | 3.09 | | | | | + | This study |
| 8 | Aeruginosamide | Ag B | 575.2678 [M+H]+ | $C_{32}H_{39}N_4O_4S$ | 0.43 | 3.55 | | + | + | | + | [49] |
| 8 | Aeruginosamide | Ag 590 | 591.2635 [M+H]+ | $C_{32}H_{38}N_4O_5S$ | −0.28 | 3.08 | | | | | + | This study |
| 8 | Aeruginosamide | Ag 661 | 660.3217 [M+H]+ | $C_{36}H_{46}N_5O_5S$ | 0.08 | 3.16 | | | | | + | This study |
| 8 | Aeruginosamide | Ag C | 674.3357 [M+H]+ | $C_{37}H_{47}N_5O_5S$ | 0.79 | 3.85 | | | | | + | [49] |
| 8 | Aeruginosamide | Ag 689 | 690.3328 [M+H]+ | $C_{37}H_{47}N_5O_6S$ | 1.18 | 3.56 | | | | | + | This study |
| 9 | Anabaenopeptin | AP D | 828.4298 [M+H]+ | $C_{44}H_{57}N_7O_9$ | −0.06 | 3.91 | | | | + | + | [57] |
| 9 | Anabaenopeptin | AP 850 | 851.4453 [M+H]+ | $C_{46}H_{58}N_8O_8$ | −0.10 | 4.97 | | | | + | + | This study |
| 9 | Anabaenopeptin | AP 857 | 858.4381 [M+H]+ | $C_{45}H_{59}N_7O_{10}$ | −1.82 | 3.44 | | | | + | + | [57] |
| 9 | Anabaenopeptin | FA A | 867.4378 [M+H]+ | $C_{46}H_{58}N_8O_9$ | −1.44 | 3.93 | | | | + | + | [57] |
| 9 | Anabaenopeptin | FA B | 881.4552 [M+H]+ | $C_{47}H_{60}N_8O_9$ | 1.13 | 4.18 | | | | + | + | [57] |
| 9 | Anabaenopeptin | AP 882 | 883.4334 [M+H]+ | $C_{46}H_{58}N_8O_{10}$ | −1.66 | 3.46 | | | | + | + | This study |
| 10 | Aeruginosin | As 722 | 643.2986 [M+H-SO3]+ | $C_{32}H_{43}N_6O_9SCl$ | −1.38 | 2.60 | | | | + | | This study |
| 10 | Aeruginosin | As 688 | 689.2947 [M+H]+ | $C_{32}H_{44}N_6O_9S$ | −1.34 | 2.50 | | | | + | | This study |
| 11 | Aeruginosin | As 650 | 651.3141 [M+H]+ | $C_{33}H_{42}N_6O_6$ | 0.70 | 2.24 | | + | | | | This study |
| 11 | Aeruginosin | As 684 | 685.2750 [M+H]+ | $C_{33}H_{41}N_6O_6Cl$ | 0.63 | 2.34 | | + | | | | This study |
| 11 | Aeruginosin | As 734 | 717.3002 [M+H-H2O]+ | $C_{29}H_{46}N_6O_7Cl_2$ | 0.98 | 2.44 | | + | | | | This study |
| 12 | Cyanobactin | Md A | 583.1901 [M+H]+ | $C_{26}H_{30}N_8O_4S_2$ | 0.85 | 3.15 | | | + | | | [35] |
| 12 | Cyanobactin | Md 584 | 585.2058 [M+H]+ | $C_{26}H_{32}N_8O_4S_2$ | −0.03 | 2.96 | + | | + | | | This study |

| | | | | | | | | | | | | |
|---|---|---|---|---|---|---|---|---|---|---|---|---|
| 12 | Cyanobactin | Md 586 | 587.2216 [M+H]$^+$ | $C_{26}H_{34}N_8O_4S_2$ | −0.20 | 3.84 | + | | + | | | This study |
| 13 | Cyanobactin | Ac C | 517.2235 [M+H]$^+$ | $C_{24}H_{32}N_6O_5S$ | 1.42 | 5.85 | + | + | | | | [35] |
| 13 | Cyanobactin | Ac 518 | 519.2385 [M+H]$^+$ | $C_{24}H_{34}N_6O_5S$ | −0.89 | 5.77 | | + | | | | This study |
| 13 | Cyanobactin | Ac B | 533.2006 [M+H]$^+$ | $C_{24}H_{32}N_6O_4S_2$ | 1.27 | 5.82 | | + | | | | [53] |
| 13 | Cyanobactin | Ac A | 535.2161 [M+H]$^+$ | $C_{24}H_{34}N_6O_4S_2$ | 0.98 | 5.78 | + | + | | | | [53] |
| | Cyanobactin | Ac D | 587.1562 [M+H]$^+$ | $C_{26}H_{30}N_6O_4S_3$ | −0.37 | 5.12 | | + | | | | [35] |
| 14 | Not defined | Unk 1448 | 725.3962 [M+2H]$^{2+}$ | $C_{73}H_{106}N_{15}O_{14}S$ | 1.02 | 5.38 | + | | | | + | This study |
| 14 | Not defined | Unk 1461 | 731.4159 [M+2H]$^{2+}$ | $C_{68}H_{114}N_{15}O_{18}S$ | −0.92 | 5.81 | + | | | | + | This study |
| 14 | Not defined | Unk 1462 | 732.4059 [M+2H]$^{2+}$ | $C_{70}H_{110}N_{16}O_{16}S$ | −2.16 | 5.50 | + | | | | + | This study |
| 14 | Not defined | Unk 1474 | 738.4238 [M+2H]$^{2+}$ | $C_{69}H_{116}N_{15}O_{18}S$ | −0.75 | 5.81 | + | | | | + | This study |
| 14 | Not defined | Unk 1476 | 739.4133 [M+2H]$^{2+}$ | $C_{71}H_{112}N_{16}O_{16}S$ | −1.42 | 5.61 | + | | | | + | This study |
| 14 | Not defined | Unk 1488 | 745.4307 [M+2H]$^{2+}$ | $C_{70}H_{118}N_{15}O_{18}S$ | −2.07 | 5.91 | + | | | | + | This study |
| 14 | Not defined | Unk 1490 | 746.4202 [M+2H]$^{2+}$ | $C_{72}H_{114}N_{16}O_{16}S$ | −1.69 | 5.56 | + | | | | + | This study |
| 14 | Not defined | Unk 1504 | 753.4296 [M+2H]$^{2+}$ | $C_{73}H_{116}N_{16}O_{16}S$ | −1.95 | 5.69 | + | | | | + | This study |
| 14 | Not defined | Unk 1508 | 755.4265 [M+2H]$^{2+}$ | $C_{73}H_{118}N_{15}O_{15}S_2$ | 0.67 | 5.41 | + | | | | + | This study |
| 14 | Not defined | Unk 1522 | 762.4341 [M+2H]$^{2+}$ | $C_{74}H_{120}N_{15}O_{15}S_2$ | 0.74 | 5.57 | + | | | | + | This study |
| 15 | Not defined | Unk 676 | 659.3628 [M+H-H$_2$O]$^+$ | $C_{30}H_{48}N_{10}O_8$ | −0.11 | 5.75 | | + | | + | + | This study |
| 15 | Not defined | Unk 678 | 661.3791 [M+H-H$_2$O]$^+$ | $C_{30}H_{50}N_{10}O_8$ | 0.12 | 5.94 | | + | | + | + | This study |
| 15 | Not defined | Unk 816 | 799.4315 [M+H-H$_2$O]$^+$ | $C_{35}H_{56}N_{14}O_9$ | −0.82 | 6.12 | | | | + | + | This study |
| 16 | Not defined | Unk 3314 | 1105.9709 [M+3H]$^{3+}$ | $C_{188}H_{247}N_{27}O_{27}$ | 2.34 | 6.05 | + | | | + | + | This study |
| 16 | Not defined | Unk 3316 | 1106.6386 [M+3H]$^{3+}$ | $C_{188}H_{249}N_{27}O_{27}$ | −0.05 | 5.84 | | | | + | + | This study |
| 16 | Not defined | Unk 3953 | 1318.7494 [M+3H]$^{3+}$ | $C_{198}H_{297}N_{41}O_{44}$ | 0.01 | 6.05 | | | | + | + | This study |
| 17 | Not defined | Unk 612 | 613.3250 [M+H]$^+$ | $C_{32}H_{44}N_4O_8$ | 2.96 | 2.95 | | | + | | | This study |
| 17 | Not defined | Unk 692 | 693.2809 [M+H]$^+$ | $C_{32}H_{44}N_4O_{11}S$ | 1.34 | 3.03 | | | + | | | This study |
| 17 | Not defined | Unk 794 | 795.2213 [M+H]$^+$ | $C_{34}H_{42}N_4O_{15}S_2$ | −0.05 | 3.00 | | | + | | | This study |
| 17 | Not defined | Unk 810 | 811.1867 [M+H]$^+$ | $C_{40}H_{35}N_4O_{13}S$ | −2.61 | 3.02 | | | + | | | This study |
| 18 | Not defined | Unk 1013 | 1014.6271 [M+H]$^+$ | $C_{56}H_{83}N_7O_{10}$ | −0.27 | 6.04 | | | | + | + | This study |
| 18 | Not defined | Unk 1029 | 1030.6211 [M+H]$^+$ | $C_{56}H_{83}N_7O_{11}$ | 0.22 | 5.91 | | | | + | + | This study |

| | | | | | | | | | | | | |
|---|---|---|---|---|---|---|---|---|---|---|---|---|
| 19 | Not defined | Unk 359 | 360.1929 $[M+H]^+$ | $C_{19}H_{25}N_3O_4$ | 2.82 | 2.33 | + | | | | | This study |
| 19 | Not defined | Unk 559 | 560.2925 $[M+H]^+$ | $C_{24}H_{41}N_5O_{10}$ | −0.21 | 2.20 | + | | | | | This study |
| 20 | Not defined | Unk 608 | 609.4340 $[M+H]^+$ | $C_{31}H_{56}N_6O_6$ | 1.00 | 6.22 | | + | | | | This study |
| 20 | Not defined | Unk 622 | 623.4494 $[M+H]^+$ | $C_{32}H_{58}N_6O_6$ | 0.64 | 6.24 | | + | | | | This study |
| 21 | Not defined | Unk 495 | 513.3036 $[M+NH_4]^+$ | $C_{23}H_{40}N_6O_7$ | 1.02 | 6.25 | | | | + | + | This study |
| 21 | Not defined | Unk 674 | 692.3845 $[M+NH_4]^+$ | $C_{25}H_{46}N_{12}O_{10}$ | −0.80 | 5.49 | + | + | + | + | + | This study |
| 22 | Not defined | Unk 669 | 670.4092 $[M+NH_4]^+$ | $C_{38}H_{51}N_7O_4$ | 2.28 | 5.62 | + | | + | | | This study |
| 22 | Not defined | Unk 831 | 832.4614 $[M+H]^+$ | $C_{44}H_{61}N_7O_9$ | 1.29 | 5.63 | + | | + | | + | This study |
| 23 | Not defined | Unk 758 | 759.3113 $[M+H]^+$ | $C_{37}H_{42}N_8O_{10}$ | 0.03 | 5.45 | | | | + | + | This study |
| 23 | Not defined | Unk 760 | 761.3274 $[M+H]^+$ | $C_{37}H_{44}N_8O_{10}$ | 2.72 | 5.66 | | + | | + | + | This study |
| 24 | Not defined | Unk 410 | 393.2088 $[M+H\text{-}H_2O]^+$ | $C_{19}H_{30}N_4O_6$ | −2.36 | 2.32 | | | | + | + | This study |
| 24 | Not defined | Unk 477 | 478.2753 $[M+H]^+$ | $C_{22}H_{35}N_7O_5$ | −1.70 | 2.17 | | | | + | + | This study |
| 25 | Not defined | Unk 334 | 335.1596 $[M+H]^+$ | $C_{17}H_{22}N_2O_5$ | −1.63 | 2.89 | | + | | + | + | This study |
| 25 | Not defined | Unk 366 | 349.1753 $[M+H\text{-}H_2O]^+$ | $C_{17}H_{26}N_4O_5$ | −2.71 | 3.55 | | | | + | + | This study |
| 26 | Not defined | Unk 516 | 534.3126 $[M+NH_4]^+$ | $C_{23}H_{40}N_4O_9$ | −0.82 | 2.28 | | | + | | + | This study |
| 26 | Not defined | Unk 560 | 578.3389 $[M+NH_4]^+$ | $C_{25}H_{44}N_4O_{10}$ | −0.16 | 2.30 | | | + | | + | This study |
| 26 | Not defined | Unk 604 | 622.3652 $[M+NH_4]^+$ | $C_{27}H_{48}N_4O_{11}$ | -0.70 | 2.32 | | | + | | + | This study |
| 27 | Not defined | Unk 946 | 947.5921 $[M+H]^+$ | $C_{46}H_{78}N_{10}O_{11}$ | −0.34 | 6.20 | + | + | | | | This study |
| 27 | Not defined | Unk 960 | 961.6081 $[M+H]^+$ | $C_{47}H_{80}N_{10}O_{11}$ | 0.07 | 6.29 | + | + | | | | This study |
| 27 | Not defined | Unk 974 | 975.6237 $[M+H]^+$ | $C_{48}H_{82}N_{10}O_{11}$ | 0.05 | 6.32 | | + | | | | This study |

Abbreviations: Ac; aerucyclamide, Ag; aeruginosamide, As; aeruginosin, AP; anabaenopeptin, Cyp; cyanopeptolin, Dha; dehydroalanine, FA; ferintoic acid, Lxx; leucine or isoleucine, MC; microcystin, Md; microcyclamide, Mg; microginin, Mv; microviridin, NP; Nosptopeptin, Unk; unknown.

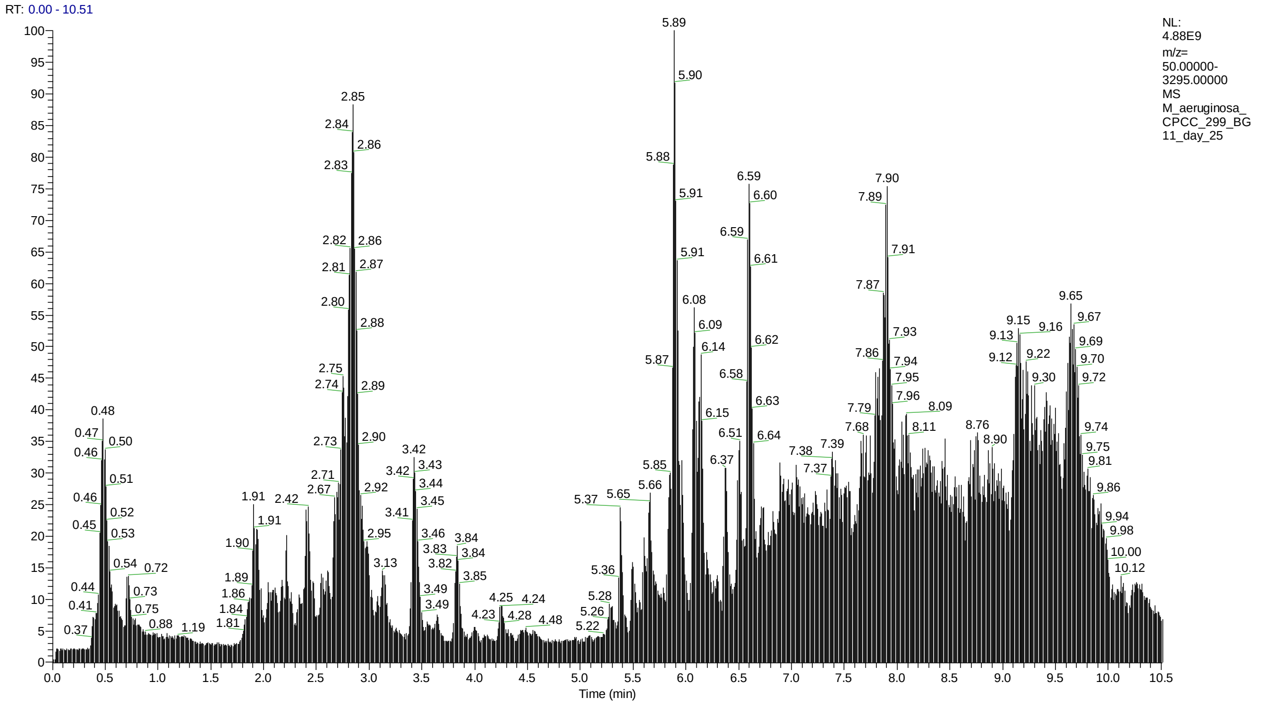


**Figure S1.** LC-MS spectrum for *M. aeruginosa* CPCC 299 grown in BG-11 medium for 25 days.

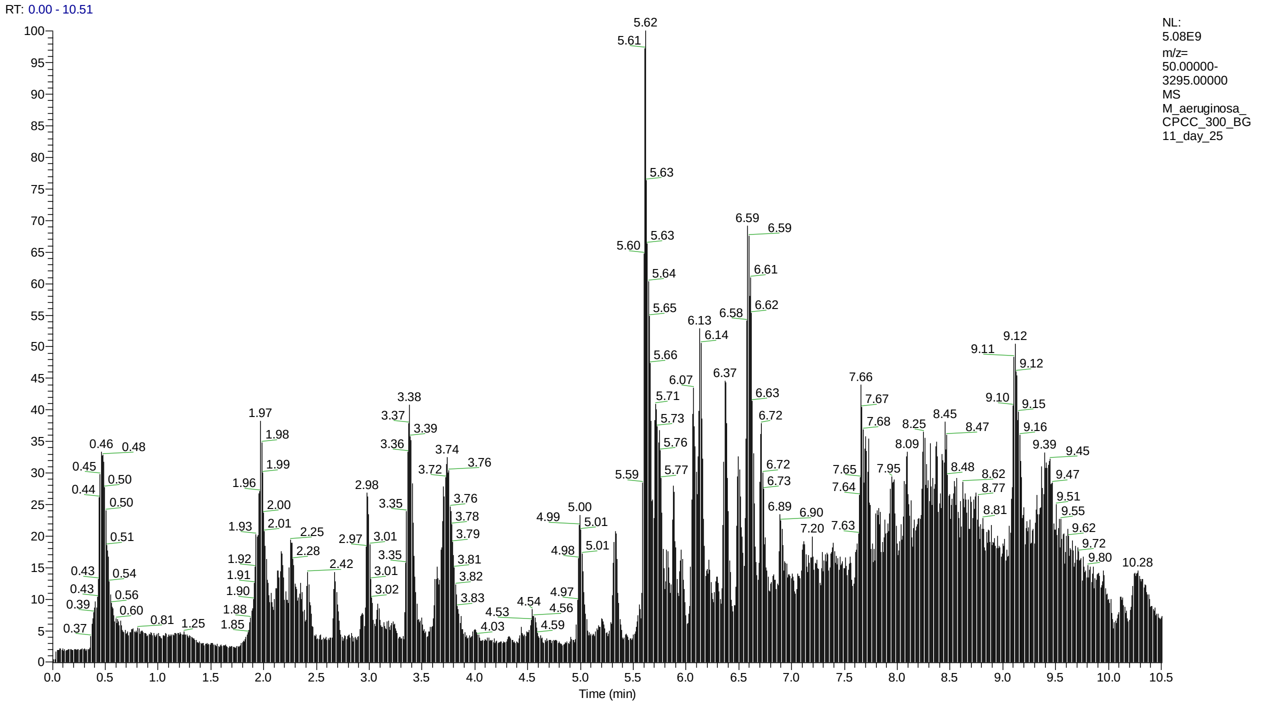


**Figure S2.** LC-MS spectrum for *M. aeruginosa* CPCC 300 grown in BG-11 medium for 25 days.

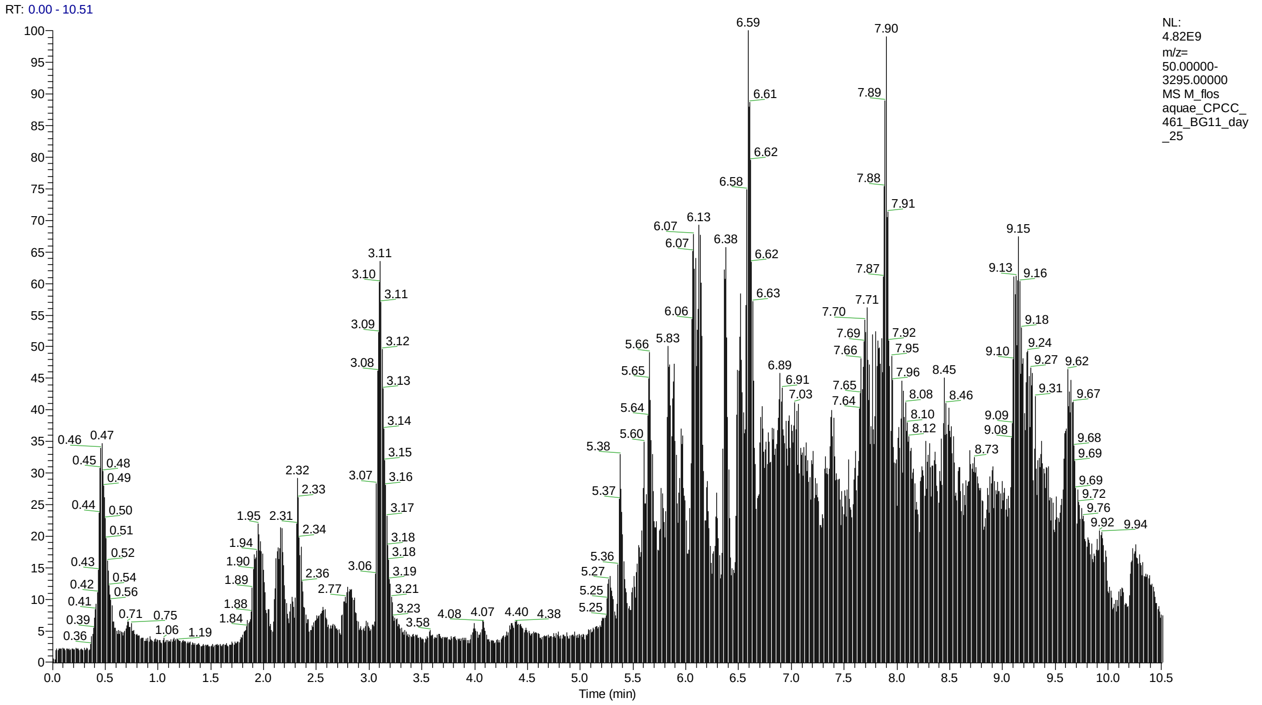


**Figure S3.** LC-MS spectrum for *M. flos-aquae* CPCC 461 grown in BG-11 medium for 25 days.

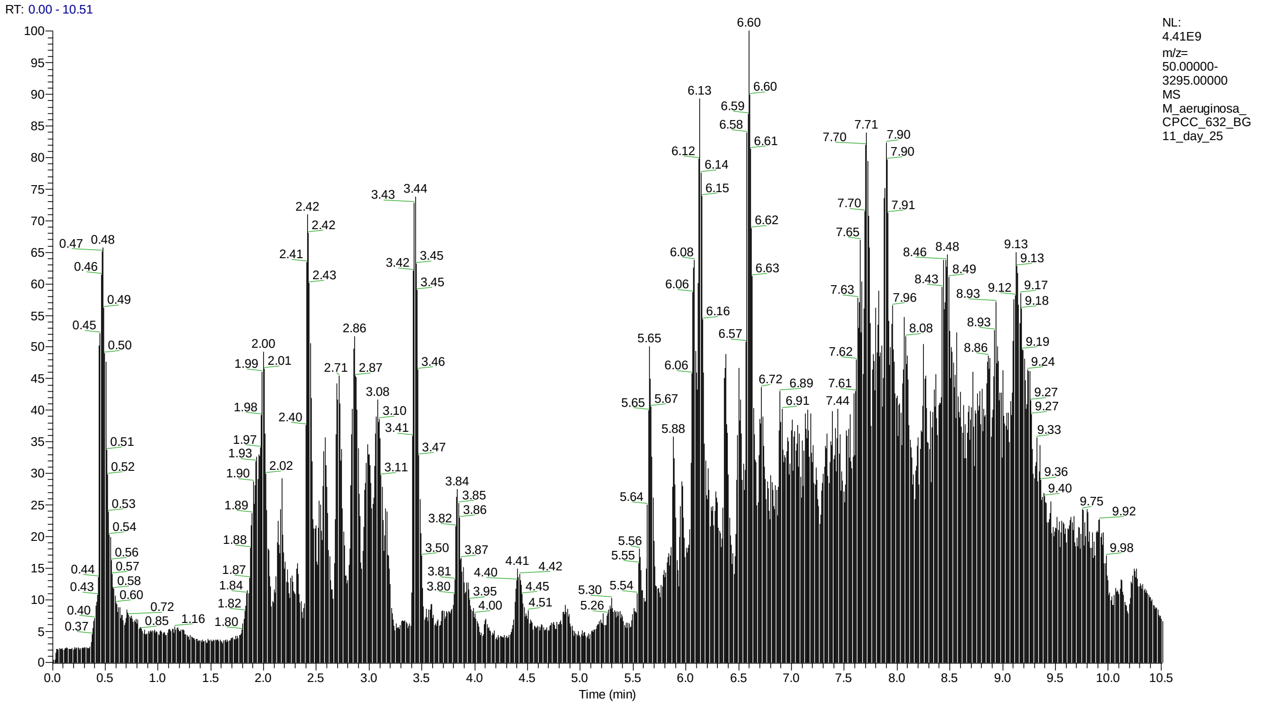


**Figure S4.** LC-MS spectrum for *M. aeruginosa* CPCC 632 grown in BG-11 medium for 25 days.

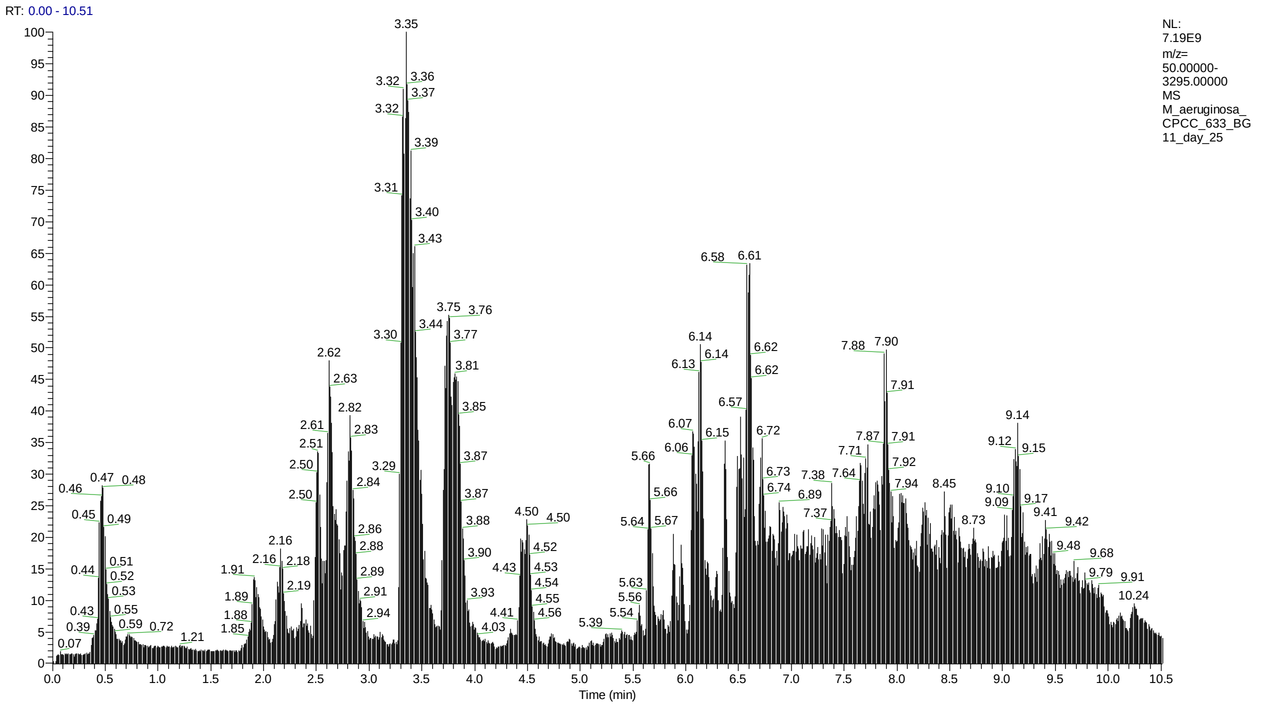


**Figure S5.** LC-MS spectrum for *M. aeruginosa* CPCC 633 grown in BG-11 medium for 25 days.

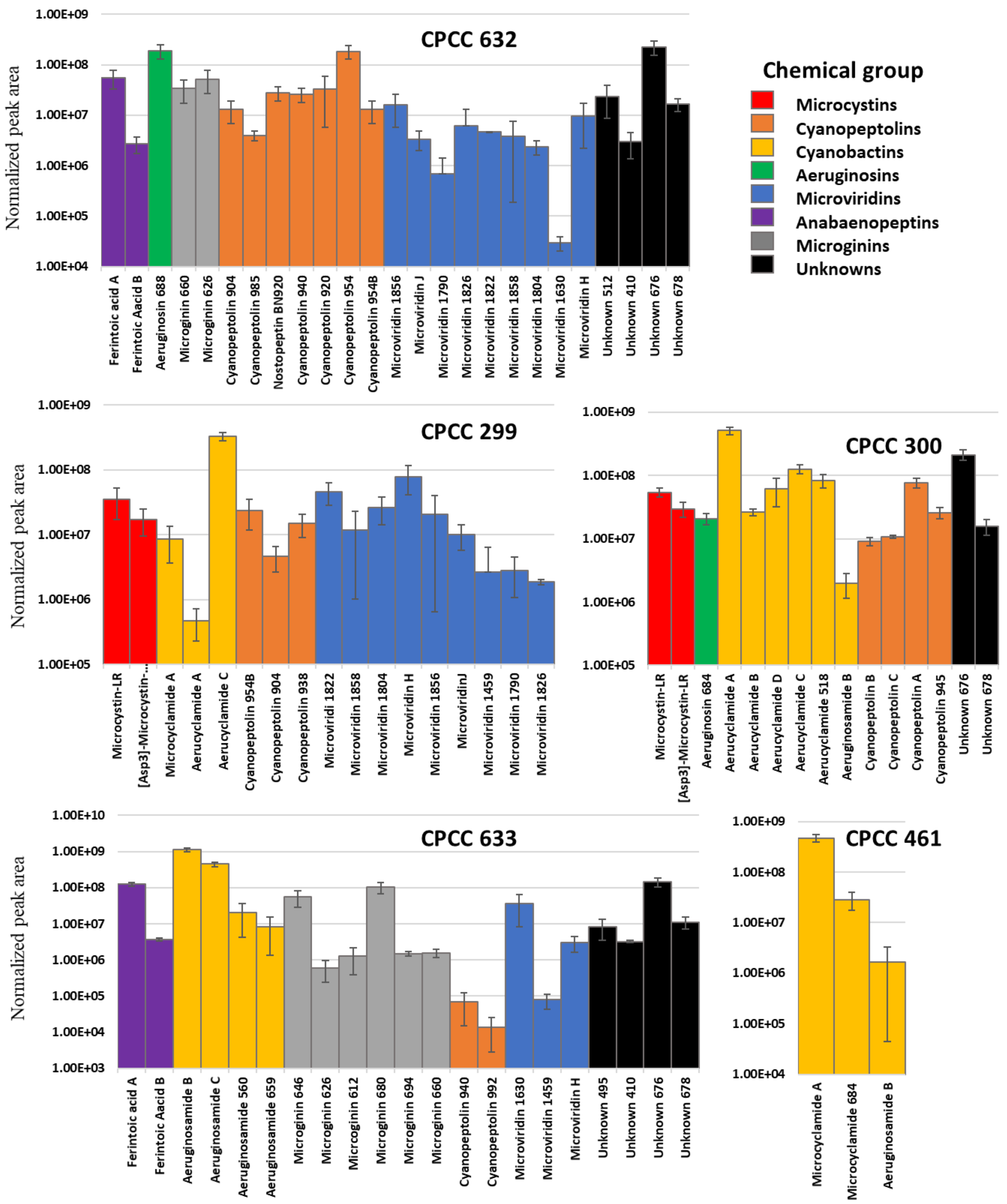


**Figure S6.** Normalized relative abundance (peak area/dry cell mass) of dominant cyanopeptides produced by five studied *Microcystis* strains included in the GNPS analysis.
